# HVC1 ameliorates hyperlipidemia and inflammation in LDLR^−/−^ mice

**DOI:** 10.1186/s12906-017-1734-z

**Published:** 2017-04-20

**Authors:** Se-Yun Cheon, Kyung-Sook Chung, Kyung-Jin Lee, Ho-Young Choi, In-Hye Ham, Dong-Hoon Jung, Yun-Yeop Cha, Hyo-Jin An

**Affiliations:** 1 College of Korean Medicine, Sangji University, Wonju-si, Gangwon-do 26339 Republic of Korea; 2 Catholic Precision Medicine Research Center, College of Medicine, The Catholic University of Korea, 222, Banpo-daero, Seocho-gu, Seoul, 06591 Republic of Korea; 3 Department of Herbology, College of Korean Medicine, Kyung hee University, Seoul, 02447 Republic of Korea; 4 Department of Rehabilitation Medicine of Korean Medicine and Neuropsychiatry, College of Oriental Medicine, Sangji University, Wonju-si, Gangwon-do 26339 Republic of Korea

**Keywords:** Hyperlipidemia, HVC1, PPAR, AMPK, Inflammatory cytokine, LDLR^−/−^

## Abstract

**Background:**

HVC1 consists of Coptidis Rhizoma (dried rhizome of *Coptischinensis*), Scutellariae Radix (root of *Scutellariabaicalensis*), Rhei Rhizoma (rhizome of *Rheum officinale*), and Pruni Cortex (cortex of *Prunusyedoensis Matsum*). Although the components are known to be effective in various conditions such as inflammation, hypertension, and hypercholesterolemia, there are no reports of the molecular mechanism of its hypolipidemic effects.

**Methods:**

We investigated the hypolipidemic effect of HVC1 in low-density lipoprotein receptor-deficient (LDLR^−/−^) mice fed a high-cholesterol diet for 13 weeks. Mice were randomized in to 6 groups: ND (normal diet) group, HCD (high-cholesterol diet) group, and treatment groups fed HCD and treated with simvastatin (10 mg/kg, *p.o.*) or HVC1 (10, 50, or 250 mg/kg, *p.o.*).

**Results:**

HVC1 regulated the levels of total cholesterol, triglyceride (TG), low-density lipoprotein (LDL) cholesterol, and high-density lipoprotein (HDL) cholesterol in mouse serum. In addition, it regulated the transcription level of the peroxisome proliferator-activated receptors (PPARs), sterol regulatory element-binding proteins (SREBP)-2, 3-hydroxy-3-methylglutaryl (HMG)-CoA reductase, lipoprotein lipase (LPL), apolipoprotein B (apo B), liver X receptor (LXR), and inflammatory cytokines (IL-1β, IL-6, and TNF-α). Furthermore, HVC1 activated 5′ adenosine monophosphate-activated protein kinase (AMPK).

**Conclusion:**

Our results suggest that HVC1 might be effective in preventing high-cholesterol diet-induced hyperlipidemia by regulating the genes involved in cholesterol and lipid metabolism, and inflammatory responses.

## Background

Hyperlipidemia is defined as increased levels of cholesterol, cholesterol esters, phospholipids, and triglycerides in the serum [1]. About 20–25% of total daily cholesterol production occurs in the liver, which is also the organ that regulates cholesterol homeostasis. Cholesterol synthesis in the liver is responsive to external factors and hence, suppressed by an increase in dietary cholesterol. Triglycerides and cholesterol both from diet and synthesized in the liver are solubilized in lipoproteins [2]. These lipoproteins contain triglyceride lipid droplets and cholesteryl esters surrounded by polar phospholipids and proteins identified as apolipoproteins. There are several types of lipoproteins in the blood. In order of increasing density, they are chylomicrons, very-low-density lipoprotein (VLDL), low-density lipoprotein (LDL), intermediate-density lipoprotein (IDL), and high-density lipoprotein (HDL). Lower protein/lipid ratios yield less dense lipoproteins. The cholesterol within different lipoproteins is identical although some is carried as “free” alcohol and others as fatty acyl esters, known also as cholesterol esters. Low-density lipoprotein receptors (LDLR) regulate cholesterol-rich IDL and LDL in serum; therefore, LDLR deficiency is related to elevated cholesterol levels, particularly IDL/LDL cholesterol [3]. LDLR^−/−^ mice demonstrate a human-like lipoprotein profile characterized by elevated cholesterol levels [4].

Peroxisome proliferator-activated receptors (PPARs) family is nuclear receptors superfamily that regulates in energy homeostasis and metabolic function such as β-oxidation of fatty acids, cholesterol metabolism [5]. The PPAR subfamily is composed of three members including PPAR-α, PPAR-δ, and PPAR-γ [6]. PPAR-α is known to regulation of genes involved in fatty acid oxidation, gluconeogenesis, cholesterol catabolism, and lipoprotein metabolism [7, 8]. Ligands for PPAR-α reduce triglycerides and LDL-cholesterol, and increase HDL-cholesterol and cholesterol efflux by inducing the expression of LXR [9, 10]. PPAR-δ regulates the expression of oxidative enzymes, and metabolic activity in the skeletal muscles, liver, and heart [11]. In addition, PPAR-δ may improve blood lipids and lipoproteins in atherosclerotic condition [12]. PPAR-δ agonist suppressed atherosclerotic lesion by improving the serum lipoprotein profiles [13]. Some studies have indicated that PPARs regulate the factors involved in fatty acid oxidation in the liver, and that PPAR-γ plays an important role in lipogenesis [14–16]. PPAR-γ is involved in the expression of lipogenic enzymes [17]. The herbal materials in HVC1, namely Coptidis Rhizoma (CR, rhizome of *Coptischinensis*), Scutellariae Radix (SR, radix of *Scutellariabaicalensis*), Rhei Rhizoma (RR, rhizome of *Rheum officinale*), and Pruni Cortex (PC, cortex of *Prunusyedoensis Matsum*are used as traditional medicines. The main bioactive compounds of CR are coptisine which improved obesity-related inflammatory response in syrian golden hamsters [18]. CR extract is reported to reduce oxidative stress and cholesterol levels [19] and the bioflavonoids from SR suppress the level of serum lipid [20] and RR possess hypolipidemic effects in hyperlipidemic rat model [21]. Among the components of HVC1, PC is reported to effective in cough, urticarial, dermatitis, and asthma [22, 23]. In addition, in our previous study, we found that prunetin, a component of Pruni Cortex, indicated anti-obesity effect via suppressing of adipogenesis [24].

In this study, we investigated HVC1’s potential to suppress high-cholesterol diet (HCD)-induced hyperlipidemia, and explored the possible molecular mechanism involved in the attenuation of lipid metabolism and hepatic inflammation.

## Methods

### Reagents

Monoclonal antibodies were purchased from Santa Cruz Biotechnology (California, USA). Oligonucleotide primers were purchased from Bioneer (Daejeon, Republic of Korea). All other reagents were purchased from Sigma-Aldrich (St. Louis, MO, USA).

### Preparation of HVC1

Pruni Cortex and Rhei Rhizome were purchased from Dongwoodang Co., Ltd. (Yeongcheon, Kyungpook, Republic of Korea). Coptidis Rhizoma and Scutellariae Radix were purchased from Dong Yang Herb Co., Ltd. (Seoul, Republic of Korea) [25]. The herbs were used at a ratio of 1:1:2:2 (CR 300 g: SR 300 g: PC 600 g: RR 600 g) respectively. The herbs were extracted using 30% (*v*/v) ethanol in water at 60 °C for 8 h. The extracts were filtered through a 10 μm cartridge paper, and the ethanol was removed by vacuum rotary evaporation (EYELA, Tokyo, Japan). The concentrates were freeze-dried, and the yield was calculated to be 13%. The powders were dissolved in distilled water for the experiments, and the residual powder was stored at −20 °C.

### Experimental animals

LDL^−/−^ mice (4 weeks old, male) were purchased from Daehan Biolink Co. Ltd. (Daejeon, Republic of Korea) and maintained under constant conditions (temperature, 22 ± 3 °C; humidity, 40–50%; light/dark cycle 12/12 h). Mice were adapted to the feeding conditions for 1 week and then given free access to food and tap water for 13 weeks. Mice were randomly separated into groups of 6 each (Table 1): ND (normal diet) group, HCD (high-cholesterol diet) group, and treatment groups fed HCD (D12336) and treated with simvastatin (10 mg/kg, *p.o.*) or HVC1 (10, 50, or 250 mg/kg, *p.o.*). With the exception of the ND group, all of the mice were fed a HCD. Body weight and dietary intake were recorded every week. At the last day of 13th week, the animals were fasted overnight. Blood samples were collected by cardiac puncture. The liver was excised, rinsed and directly stored at −80 °C until analyses. All procedures were conducted in accordance with the National Institute of Health guidelines and approved by the Ethical Committee for Animal Care and the Use of Laboratory Animals of Sangji University (reg.no. 2014–04).

**Table 1:** Caloric content and ingredient composition of each diet

Caloric content	Normal diet		Caloric content	HCD	
	gm%	kcal%		gm%	kcal%
Protein	20.3	20.8	Protein	21	20
Carbogydrate	66.0	57.7	Carbogydrate	46	45
Fat	11.5	11.5	Fat	16	35
Ingredient	gm	kcal	Ingredient	gm	kcal
Casein-	200	800	Casein-	75	300
L-cystine	0	0	Soy protein	130	520
Corn starch	150	600	DL-Methionine	2	8
Maltodextrin10	0	0	Corn starch	275	1100
			Maltodextrin	150	600
Sucrose	500	2000	Sucrose	30	120
Cellulose, BW200	50	0	Cellulose, BW200	90	0
Soybean oil	0	0	Soybean oil	50	450
Corn oil	50	450	CoCoa Butter	75	675
Lard	0	0	CoConut Oil, 76	35	315
Mineral Mix S10001	35	0	Mineral Mix S10001	35	0
Calcium Carbonate	0	0	Calcium Carbonate	5.5	0
Dicalcium phosphate	0	0	Sodium Chloride	8	0
Potassium Citrate	0	0	Potassium Citrate	10	0
Vitamin Mix V10001	10	40	Vitamin Mix V10001	10	40
Choline Bitartrate	2	0	Choline Bitartrate	2	0
			Cholesterol	12.5	0
			Sodium Cholic Acid	5	0
			FD&C Red Dye #40	0.1	0

### Histological examination

To analysis of atherosclerosis, aorta roots were frozen in embedding media for Oil red O staining analysis. The aorta roots were sectioned at a thickness of 7 μm at −20 °C by using a CM3050 cryostat (Leica, Wetzlar, Germany). The slides were fixed and stained with Oil red O dye (sigma). After staining, the slides were washed three times with 1, 2-propranediol (85%) and then with deionized water. Images were acquired using an SZX10 microscope (Olympus, Tokyo, Japan). The fold change of Oil red O positive area was quantified using Adobe Photoshop 9.0.

### Analysis of serum lipid profiles

Blood samples were collected and centrifuged at 1003 × g, for 15 min at room temperature to obtain serum samples. Unused samples were immediately frozen at −70 °C for later measurements. Serum concentrations of total cholesterol (TC), LDL cholesterol, HDL-cholesterol and triglycerides were determined by enzymatic methods with commercial kits (BioVision, Milpitas, California, USA).

### Western blot analysis

Liver tissues were homogenized in PRO-PREP™ protein extraction solution (Intron Biotechnology, Seoul, Republic of Korea) and incubated for 20 min at 4 °C. Debris was removed by micro-centrifugation11000 xg, followed by quick freezing of the supernatants. The protein concentration was determined using the Bio-Rad protein assay reagent according to the manufacturer’s instructions (Bio-Rad, California, USA). Proteins were electro-blotted onto a polyvinylidene difluoride (PVDF) membrane following separation on a 10–12% SDS polyacrylamide gel. The membrane was incubated for 1 h with blocking solution (5% skim milk) at room temperature, followed by incubation overnight with primary antibodies including, PPAR-γ, SREPB-2, HMGCR, phosphor-AMPK, AMPK (dilution, 1:1000), β-actin (dilution, 1:2000) at 4 °C. Blots were washed three times with Tween 20/Tris-buffered saline (T/TBS) and incubated in a horseradish peroxidase-conjugated secondary antibody (dilution, 1:2500) for 2 h at room temperature. The blots were again washed three times with T/TBS, and then developed by enhanced chemiluminescence (GE Healthcare, Wisconsin, USA).

### RNA separation and quantitative real-time PCR (qRT-PCR) analysis

The liver tissues were homogenized, and total RNA was isolated using a Trizol reagent (Invitrogen, Carlsbad, California, USA). cDNA was obtained using isolated total RNA (1 μg), d(T)16 primer, and AMV reverse transcriptase. Relative gene expression was quantified using real-time PCR (Real Time PCR System 7500, Applied Biosystems, California, USA) with SYBR green PCR master mix (Applied Biosystems). The gene Ct values of PPARs, HMGCR, SREBP-2, apoB, LPL, LXR and inflammatory cytokines were normalized using gene express 2.0 program (Applied Biosystems) to the Ct value of GAPDH. Oligonucleotide primers were purchased from Bioneer (Deajeon, Republic of Korea) (Table 2). The total reaction volume was 20 μl and consisted of 1 μl cDNA, 0.4 μl of primers, 10 μl SYBR green master mix and 8.2 μl DEPC treated water (Intron Biotechnology, Seoul, Republic of Korea).

**Table 2:** Primer sequences

Gene	Primer sequences 5′-3’	
PPARγ	forward	TTGGAATCAGCTCTGTGCA
	reverse	CCATTGGGTCAGCTCTTGTG
PPARα	forward	ACGATGCTGTCCTCCTTGATG
	reverse	GTGTGATAAAGCCATTGCCGT
PPARδ	forward	AGATGGTGGCAGAGCTATGACC
	reverse	TCTCCTCCTGTGGCTGTTCC
HMG-CR	forward	TGGCAGAAAGAGGGAAAGG
	reverse	CGCCTTTGTTTTCTGGTTGA
apoB	forward	TCACCATTTGCCCTCAACCTAA
	reverse	GAAGGCTCTTTGGAAGTGTAAAC
LPL	forward	TGGAGAAGCCATCCGTGTG
	reverse	TCATGCGAGCACTTCACCAG
SREBP-2	forward	TGCTGGATGACGCAAAGGTC
	reverse	AAAGGAGAGGCCCAGGAAGG
LXR	forward	TCCTACACGAGGATCAAGCG
	reverse	AGTCGCAATGCAAAGACCTG
TNFα	forward	CCAGTGTGGGAAGCTGTCTT
	reverse	AAGCAAAAGAGGAGGCAACA
IL-6	forward	CTGCAAGAGACTTCCATCCAGTT
	reverse	GAAGTAGGGAAGGCCGTGG
IL-1β	forward	GATCCACACTCTCCAGCTGCA
	reverse	CAACCAACAAGTGATATTCTCCATG
GAPDH	forward	GACGGCCGCATCTTCTTGT
	reverse	CACACCGACCTTCACCATTTT

### Statistical analysis

Data are expressed as mean ± standard deviation (SD) of triplicate experiments. Statistically significance was determined using ANOVA and Dunnett’s post hoc test, and *P*-values of less than 0.05 were considered statistically significant.

## Results

### Effects of HVC1 on plaque formation and serum lipid profiles in HCD-fed LDLR^−/−^ mice

**Fig. 1:**
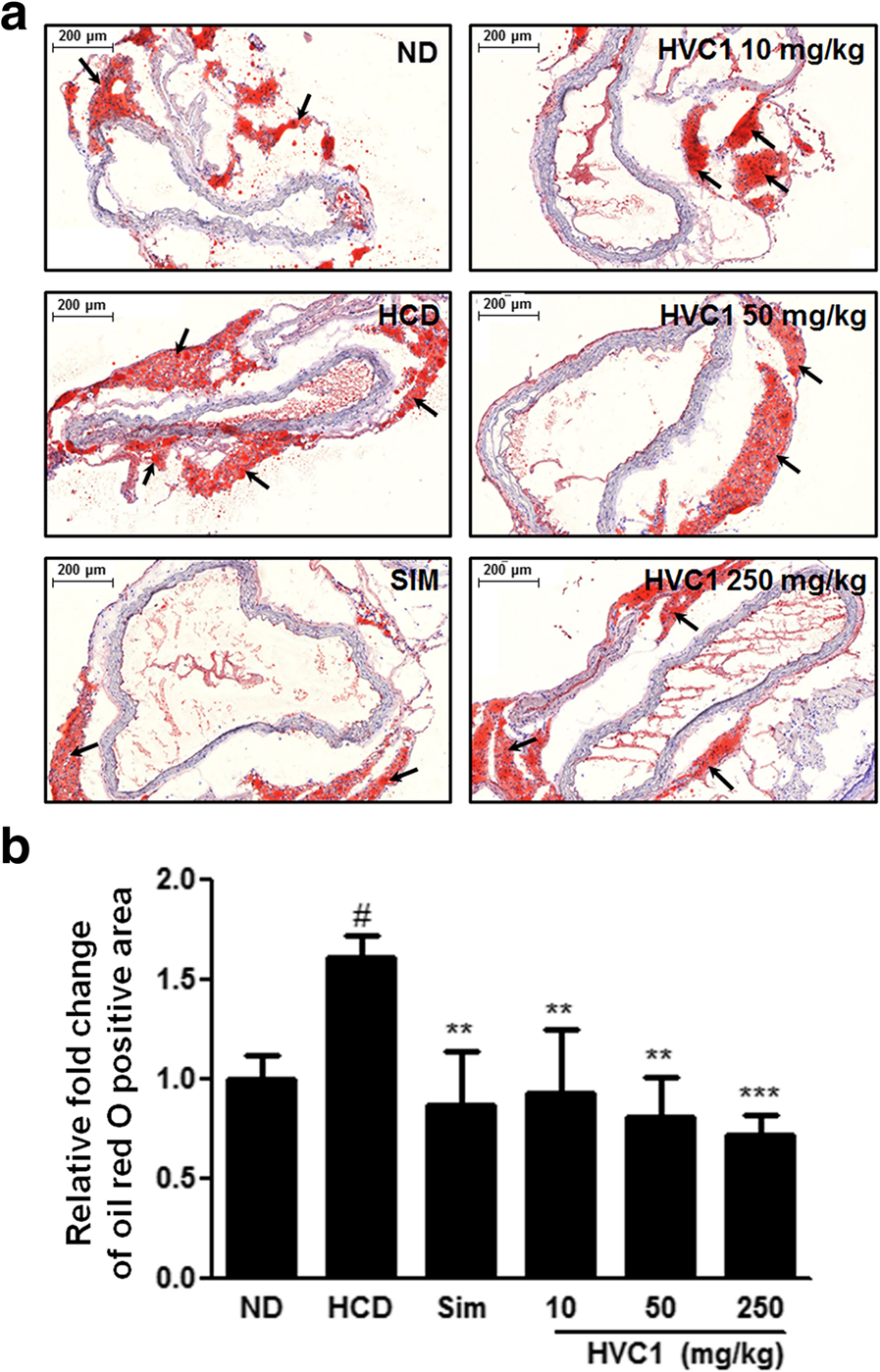
Effect of HVC1 on plaque formation in LDLR^−/−^ mice fed a HCD. (**a**) Representative images of the aortic root and (**b**) quantitative analysis of Oil *red* O positive area. Oil *red* O staining was performed to show lipid in aortic root (*black arrows*, original magnification × 100). The fold change of Oil *red* O positive area was quantified using Adobe Photoshop 9.0. ND: Normal diet group; HCD: High-cholesterol diet group; Sim: Simvastatin (10 mg/kg) treated with HCD group; HVC1: HVC1 treated with HCD group. The values represent mean ± S.D. (significant as compared to HCD, ^**^ *p* < 0.01, ^***^ *p* < 0.001, significant as compared to ND, ^#^ *p* < 0.05)

To investigate the effect of HVC1 on atherosclerosis, we examined Oil red O-stained lesions in the aortic roots. The plaque formation indicated as relative fold change of Oil red O positive area in HCD-fed LDLR^−/−^ mice was almost 1.6 times higher than that in the ND group. In contrast to the HCD group, HVC1 administration significantly suppressed the plaque formation in dose-dependent manner in LDLR^−/−^ mice fed HCD (Fig. 1a and b).

**Fig. 2:**
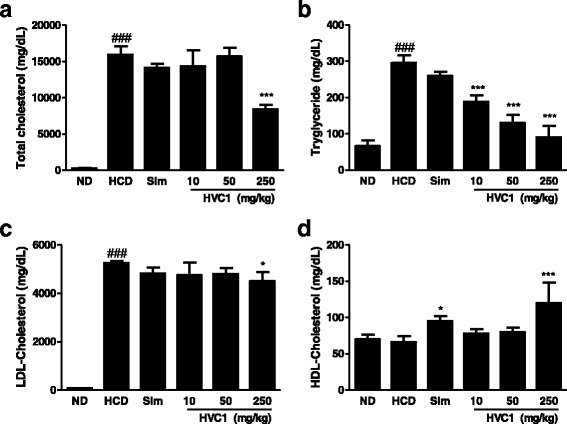
Effect of HVC1 on the serum lipid profile in LDLR^−/−^ mice fed a HCD. Total-cholesterol (**a**), triglyceride (**b**), LDL-cholesterol (**c**), HDL-cholesterol (**d**). ND: Normal diet group; HCD: High-cholesterol diet group; Sim: Simvastatin (10 mg/kg) treated with HCD group; HVC1: HVC1 treated with HCD group. The values represent mean ± S.D. (significant as compared to HCD, ^*^ *p* < 0.05, ^***^ *p* < 0.001, significant as compared to ND, ^###^ *p* < 0.001)

After 13 weeks on HCD, the serum levels of TC, TG, and LDL-cholesterol in the HCD group were significantly higher than those in any other group (Fig. 2a-c). However, serum TC, TG and LDL-cholesterol levels in the HVC1-treated group (250 mg/kg, *p.o.*) significantly decreased compared to that in the HCD group. In addition, the HVC1 treated group (250 mg/kg) had increased levels of HDL-cholesterol when compared with the HCD group (Fig. 2d).

### Inhibitory effects of HVC1 on expression of PPARs in HCD-fed LDLR^−/−^ mice

**Fig. 3:**
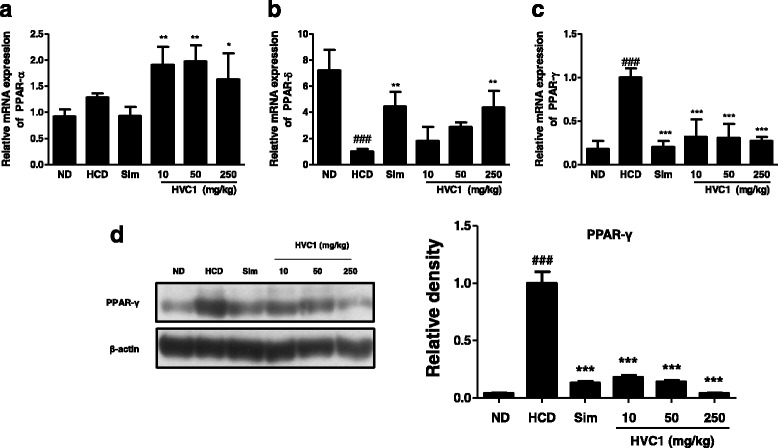
Inhibitory effect of HVC1 on the HCD-induced PPARs expressions in LDLR^−/−^ mice. LDLR^−/−^ mice were randomized into the ND group, HCD group, and treatment groups fed HCD with simvastatin (10 mg/kg) or HVC1 (10, 50 or 250 mg/kg) for 13 weeks. mRNA levels of PPARs (**a**-**c**) were analyzed by real-time PCR analysis. Expressions of PPAR-γ protein was determined by western blot assay using specific anti-PPAR-γ antibody (**d**). β-actin was used as a loading control. Values represent mean ± S.D. of three independent experiments (significant as compared to HCD, ^*^ *p* < 0.05, ^**^ *p* < 0.01, ^***^ *p* < 0.001, significant as compared to ND, ^###^ *p* < 0.001)

The effects of HVC1 on the mRNA expression of PPAR family members in liver tissue were examined by qRT-PCR analysis. As shown in Fig. 3, the mRNA expression of PPAR-α in the HCD group was not different from that in the ND group. However, in the HVC1 groups (10, 50, and 250 mg/kg, *p.o.*), the mRNA expression of PPAR-α significantly increased compared with the HCD group. In addition, administration of HVC1 (250 mg/kg, *p.o.*) increased the mRNA expression levels of PPAR-δ compared to that in the HCD group, while in the HCD group, PPAR-δ levels significantly decreased compared with the ND group. Conversely, the mRNA expression of PPAR-γ in the HVC1 group significantly decreased compared to that in the HCD group while being significantly higher in the HCD group than in the ND group. Since PPAR-γ plays an important role in adipogenesis and PPAR-γ activation results in reduction of free fatty acid (FFA) efflux and triacylglycerol synthesis [26], we examined PPAR-γ protein expression levels if the expression of PPAR-γ protein paralleled the transcription of its mRNA in LDLR^−/−^ mice. As shown in Fig. 3d, PPAR-γ protein levels increased in the HCD group relative to the ND group. Compared to the HCD group, however, the simvastatin and HVC1-treated groups exhibited marked decrease in the protein levels of PPAR-γ.

### HVC1 regulates lipid metabolism and cholesterol synthesis in HCD-fed LDLR^−/−^mice

**Fig. 4:**
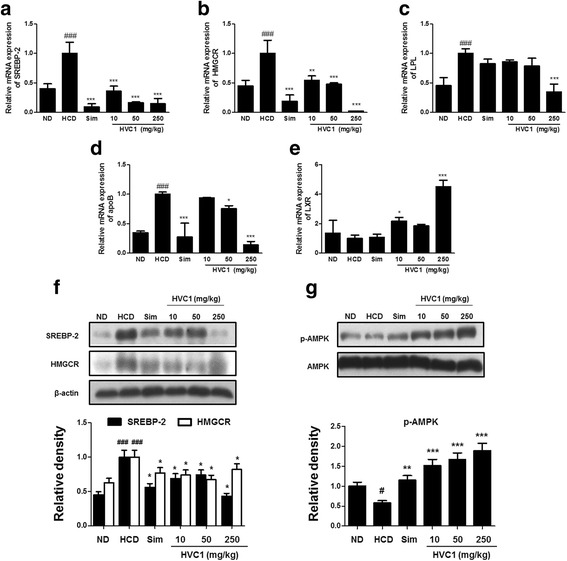
HVC1 regulation of cholesterol metabolism and lipid synthesis in LDLR^−/−^ mice. Total RNA was subjected to real-time PCR as described in the Methods section. Cholesterol and lipid metabolism-related genes mRNA levels were analyzed by real-time PCR analysis. (**a**) SREBP-2, (**b**) HMGCR, (**c**) LPL, (**d**) apoB, and (**e**) LXR. Protein levels of (**f**) SREBP-2, (**g**) HMGCR, (H) p-AMPK, and AMPK in liver tissues were analyzed by western blot. Proteins were determined by western blot assay using specific antibody. β-actin was used as a loading control. Values represent mean ± S.D. of three independent experiments (significant as compared to HCD, ^*^ *p* < 0.05, ^**^ *p* < 0.01, ^***^ *p* < 0.001, significant as compared to ND, ^###^ *p* < 0.001)

To investigate whether the lipid metabolism and biosynthesis of cholesterol in HVC1 treated mice was associated with molecular signaling by the genes involved in hyperlipidemia, we examined the expression levels of related genes that are key transcription factors in the liver. In the liver tissue, the mRNA expression levels of SREBP-2, HMGCR, LPL, and apo B significantly decreased in a dose dependent manner in the HVC1-treated groups compared to that in the HCD group. Conversely, the expression of LXR mRNA in the HVC1 groups increased compared to that in the HCD group (Fig. 4a-e). In addition, treatment with HVC1 markedly increased the expression levels of SREBP-2 and HMGCR in the HCD group compared to that in the ND group. The simvastatin and HVC1-treated groups, however, exhibited reduced expression levels of the proteins (Fig. 4f).

AMPK is a major regulator of energy metabolism, and its phosphorylation is involved in the regulation of adipocyte differentiation [27]; therefore, we investigated whether HVC1 regulated energy metabolism through the AMPK pathway. As shown in Fig. 4g, treatment with HVC1 inhibited HCD-induced dephosphorylation of AMPK in HCD-fed LDLR^−/−^ mice.

### Inhibitory effects of HVC1 on mRNA expression of inflammatory cytokines in HCD-fed LDLR^−/−^ mice

**Fig. 5:**
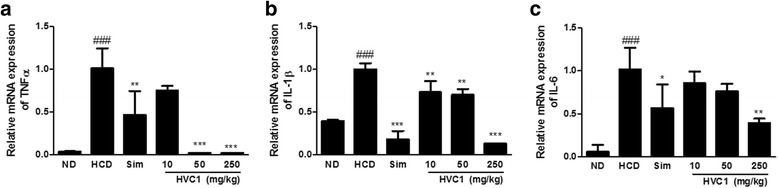
Inhibitory effects of HVC1 on mRNA expression of inflammatory cytokines in HCD induced LDLR^−/−^ mice. Inflammatory cytokine genes mRNA levels were analyzed by real-time PCR analysis. (**a**) TNF-α, (**b**) IL-1β, (**c**) IL-6.Total RNA was subjected to real-time PCR as described in the Methods section. Values represent mean ± S.D. of three independent experiments (significant as compared to HCD, ^*^ *p* < 0.05, ^**^ *p* < 0.01, ^***^ *p* < 0.001, significant as compared to ND, ^###^ *p* < 0.001)

Some studies have reported that a correlation exists between plasma total cholesterol and the development of hepatic inflammation [26, 28]. These reports led us to hypothesize that plasma TC as an important cause for the development of inflammation in HCD-fed LDLR^−/−^ mice. Therefore, we investigated the expression of genes associated with inflammation in the liver of HCD-fed LDLR^−/−^ mice. The mRNA expression levels of inflammatory cytokines in the HCD group were significantly up-regulated in comparison to those in the ND group. In contrast, HVC1 markedly reduced the mRNA expression of tumor necrosis factor (TNF)-α, interleukin (IL)-6, and IL-1β in LDLR^−/−^ mice (Fig. 5). The results suggest that HVC1 treatment may influence the HCD-induced expression of inflammatory genes in LDLR^−/−^ mice.

## Discussion

Hyperlipidemia is related to increased levels of lipids, including cholesterol and triglyceride in the plasma. Hyperlipidemia increases the risk of developing cardiovascular disease [29]. In this study, we showed the inhibitory effect of HVC1 on hyperlipidemia-related factors in HCD-fed LDLR^−/−^ mice. The effects of HVC1 included regulation of cholesterol synthesis, lipid accumulation, and levels of inflammatory cytokines in HCD-fed mice.

Increased blood lipid levels, especially LDL-cholesterol level, can promote atherosclerosis; therefore, decreasing lipid level is important in reducing atherosclerosis [30, 31]. In this study, HVC1 suppressed the serum levels of LDL-cholesterol, TG, and TC and increased HDL-cholesterol (Fig. 2). These results suggest that HVC1 is crucial for reducing the risk of hyperlipidemia.

PPARs are members of the nuclear hormone receptor superfamily, and they regulate various physiological functions, such as glucose and lipid homeostasis, inflammatory responses, cell differentiation [5]. Lee et al. have reported that PPAR-α and-γ agonists (WY14643 and rosiglitazone) decreased hyperlipidemia by increasing the protein expression of malonyl-CoA decarboxylase (MCD) [32]. PPAR-δ agonists are also reported to have a role in lipid metabolism and they improve metabolic syndrome. They have been noted to enhance HDL cholesterol and decrease LDL cholesterol in insulin-resistant obese monkeys [33]. PPAR-γ plays a role in hyperlipidemia, triglyceride clearance and hepatic steatosis [27, 34]. Some studies reported that HCD administration lead to hepatic damage such as hepatic steatosis in mice [35, 36] and PPAR-γ act as a key modulator of high-fat diet-induced liver steatosis [37, 38]. In this study, we found that HVC1 enhanced the mRNA expression levels of PPAR-α and δ, while HVC1 significantly decreased PPAR-γ mRNA and protein expression levels in liver which is induced to hepatic steatosis. These results suggested that modulation of PPARs expression might be one of the mechanisms by which HVC1attenuates lipid accumulation in hyperlipidemia and hepatic steatosis affected by HCD.

The liver plays a central role in lipid metabolism. Some orphan nuclear hormone receptors such as LXR, retinoid X receptor (RXR), farnesoid X receptor (FXR), and PPAR are related to genes involved in cholesterol metabolism [39, 40]. LXR plays a key important role in cholesterol sensor. LDLR^−/−^ mice have functionally disordered bile acid production, which leads to cholesterol ester accumulation [41]. In addition, SREBP is a transcription factor that regulates the biosynthesis of cholesterol and fatty acids [42]. The precursor of SREBP is synthesized in the endoplasmic reticulum (ER) membrane-bound protein. It is activated, cleaved, and then translocated to the nucleus. SREBP-2 promotes the expression of target genes involved in cholesterol biosynthesis such as 3-hydroxy-3-methylglutaryl-CoA synthase (HMGCS), HMGCR and LDLR [43, 44]. HMGCR is a transmembrane protein that is associated with the biosynthesis of cholesterol [45, 46]. In this study, HVC1 decreased the mRNA expression levels of SREBP-2, HMGCR, LPL, and apoB and increased that of LXR in the liver tissue of HCD-fed LDLR^−/−^ mice. In addition, HVC1 markedly increased the protein expression level of SREBP-2 and HMGCR. These data suggested that HVC1 regulated lipid synthesis-related markers through the modulation of adipogenic gene related to cholesterol metabolism.

AMPK is a complex of α/β/γ subunits, which regulate lipid and carbohydrate metabolism, immune response, cell growth, and protein synthesis [47, 48]. AMPK plays an important role in the regulation of fatty acid oxidation by the phosphorylation and inactivation of acetyl-CoA carboxylase (ACC) [49]. It also plays a central role in lipid metabolism in the liver. Hepatic ACC has been found to be regulated in the liver [50]. In addition, AMPK is a key enzyme in cholesterol synthesis via regulation HMGCR [51]. Therefore, AMPK regulates fatty acid oxidation and cholesterol synthesis. The activation of AMPK, results in suppression of lipogenesis in the liver, which inhibits lipid accumulation [52, 53] and AMPK phosphorylation is inhibited in mice fed a HCD [27]. In this study, HVC1 significantly increased AMPK phosphorylation resulting in regulation of lipid metabolism-related genes in HCD-fed LDLR^−/−^mice (Fig. 4). These findings suggested that hypolipidemic effects of HVC1 could dependent on AMPK pathway in HCD-fed LDLR^−/−^mice.

Hyperlipidemia not only involves elevation in serum lipids, but it is also an inflammatory disease, as excessive lipid accumulation has been known to trigger local inflammatory reactions [54, 55]. The inflammatory processes mostly coincide with increased local fat accumulation as observed in nonalcoholic steatohepatitis [14]. In addition, inflammatory processes occur during the cardiovascular disease process [33, 56, 57]. Blockade of inflammatory cytokines has been shown to reduce the incidence of cardiovascular disease [29]. In this study, our results showed that HVC1 significantly reduced the mRNA expression levels of inflammatory cytokines (TNF-α, IL-1β, and IL-6) in HCD-fed LDLR^−/−^ mice. Many cytokines participated in the development of atherosclerotic leading to plaque formation [58]. Expression of IL-1-family members and their receptors has been demonstrated in atherosclerotic plaques. Furthermore, inhibition of TNF-α reduces atherosclerosis in apolipoprotein E knockout mice [59]. In these regards, we could suggest that inhibitory effects of HVC1 on mRNA expression levels of inflammatory cytokines are related to the development of atherosclerotic plaque.

## Conclusions

We demonstrated that HVC1 has an inhibitory effect of hyperlipidemia involving inflammation in HCD-fed LDLR^−/−^ mice. Our findings indicated that HVC1 treatment could suppress the development of hyperlipidemia via regulation of cholesterol metabolism and inflammatory processes as observed in the HCD-fed LDLR^−/−^ mice. Therefore, HVC1 may be used for the prevention or treatment of hyperlipidemia.

## Abbreviations

LDLR^−/−^: Low-density lipoprotein receptor-deficient
TG: Triglyceride
LDL: Low-density lipoprotein
HDL: High-density lipoprotein
PPARs: Peroxisome proliferator-activated receptors
SREBP-2: Sterol regulatory element-binding proteins
HMG-CoA reductase: 3-hydroxy-3-methylglutaryl
LPL: Lipoprotein lipase
apo B: Apolipoprotein B
LXR: Liver X receptor
AMPK: 5′ adenosine monophosphate-activated protein kinase
HCD: High-cholesterol diet
FFA: Free fatty acid
TNF: Tumor necrosis factor
IL: Interleukin
MCD: Malonyl-CoA decarboxylase
ACC: Acetyl-CoA carboxylase
